# Association of neck circumference and cognitive impairment among Chinese elderly

**DOI:** 10.1002/brb3.937

**Published:** 2018-02-14

**Authors:** Jin‐Mei Chen, Qing‐Wei Li, Guo‐Xin Jiang, Shu‐Jun Zeng, Jun Shen, Ji Sun, Dan‐Hong Wu, Qi Cheng

**Affiliations:** ^1^ Department of Neurology Shanghai Ninth People's Hospital Shanghai Jiao Tong University School of Medicine Discipline Construction Research Center of China Hospital Development Institute Shanghai Jiao Tong University Shanghai China; ^2^ School of Public Health Shanghai Jiao Tong University Shanghai China; ^3^ Department of Psychiatry Tongji Hospital Tongji University School of Medicine Shanghai China; ^4^ Shanghai Mental Health Central Shanghai Jiao Tong University School of Medicine Shanghai China; ^5^ Department of Public Health Sciences Karolinska Institute Stockholm Sweden; ^6^ Department of Neurology Shanghai Fifth People's Hospital Fudan University Shanghai China

**Keywords:** cognitive impairment, elderly, interaction, neck circumference, risk factor

## Abstract

**Objectives:**

To investigate the association between neck circumference (NC) and cognitive impairment and interactions between relevant variables to the risk of cognitive impairment.

**Methods:**

A population‐based survey was conducted among elderly inhabitants aged 60 years and over from a community in Shanghai suburb. Multivariate logistic regression analyses were performed to evaluate associations and log likelihood ratio tests to examine interactions.

**Results:**

Cognitive impairment was identified in 269 (10.8%) subjects from 2,500 participants. Higher BMI (OR = 1.55; 95% CI = 1.11–2.16), higher WHR (OR = 1.44; 95% CI = 1.07–1.95), and higher total cholesterol (TC) (OR = 1.52; 95% CI = 1.09–2.13) were significantly associated with the increased risk of cognitive impairment. Significant interactions were observed between TC and a few other relevant variables, respectively.

**Conclusions:**

NC was associated with the high risk of cognitive impairment. Additive effects of NC with TC on cognitive impairment were observed.

## 1 INTRODUCTION

Cognitive impairment is an essential part of the diagnostic criteria for dementia, and it may indicate the initiation of Alzheimer's disease or other types of dementia. Studies report a rising prevalence rate of cognitive impairment (Cui et al., 2011) and dementia (Cheng et al., 2014) among the elderly as the population ages, particularly in China. Overweight or obesity in older adults is associated with increased risk for cognitive and functional decline, and the anthropometric measures including body mass index (BMI), waist circumference (WC), and waist‐to‐height ratio (WHR) were used widely in the assessment of overweight or obesity (Kharabian et al., 2016; Maksimovic et al., 2016; Stabe et al., 2013). Epidemiological population‐based studies declared that many risk factors are related to the decline in cognitive function, such as hypertension, obesity, and diabetes (Chen, Cui, et al., 2014; Chen, Jiang, Li, Zhou, & Cheng, 2014; Lee et al., 2014; Loef & Walach, 2013). Obesity is known to be a risk factor for hypertension, diabetes, and cardiovascular disease and has multiple health implications (Loef & Walach, 2013). Recently, neck circumference (NC) was also recognized as a screening measure for identifying overweight or obese individuals in adolescents and adults (Li et al., 2014; Preis et al., 2010), as well as for making the prediction of metabolic syndrome (Mets) and obesity (Yan et al., 2014). NC was the significant independent indicator for obesity and Mets (Li et al., 2014; Stabe et al., 2013; Yan et al., 2014).The Framingham Heart Study indicated that NC might be increased due to the existence of pathogenic, fatty deposits (Preis et al., 2010). Mets and obesity were found to be associated with the risk of cognitive impairment (Ng et al., 2016; Yao, Jiang, Zhou, Chen, & Cheng, 2016). Results from those studies made it reasonable to hypothesize that NC might be associated with the risk of cognitive impairment.

Indeed, data assessing the association of NC with cognitive decline in older Chinese subjects are lacking. In this study, we conducted a cross‐sectional study to examine whether NC was associated with the risk of cognitive impairment and to explore interactions between various relevant variables to the risk of cognitive impairment among elderly in Shanghai suburb, China.

## 2 METHODS

### 2.1 Subjects

Data were obtained from a population‐based epidemiological study on cognitive impairment and dementia among elderly population, which was a cross‐sectional study. Study participants were local residents aged 60 years and over from Sheshan town in the Songjiang district, located in the southwestern suburb of Shanghai. The interviews were conducted during the period of April to May in 2015. Face‐to‐face interview, data collection, and relevant investigations were conducted by trained medical staff, and the details were described elsewhere (Chen, Cui, et al., 2014; Chen, Jiang, Li, Zhou, & Cheng, 2014; Cheng et al., 2014). Additionally, we excluded subjects with recent or ongoing infections, malignant diseases, and other serious diseases, or taking corticosteroids or immunosuppressive drugs. This study was conducted according to the guidelines laid down in the Declaration of Helsinki, and all procedures involving human subjects were approved by the Ethics Committee of Ninth Hospital affiliated to School of Medicine Shanghai Jiao Tong University.

Prior to the survey, we explained the consent process, study procedures, and purpose to the participants. Informed consent was obtained from each subject, and for participants who were illiterate or severely demented, the consent was signed by their legal guardian accompanying them to the visits. There were 2,500 individuals completed the interview in that study, and the Chinese version of the Mini‐Mental State Examination (C‐MMSE) was used to screen subjects with cognitive impairment (Cui et al., 2011; Zhang et al., 1990). Cognitive impairment was defined as subjects with the C‐MMSE score, adjusted for education level, below the cutoff points determined in previous work (Yao et al., 2010): 17/18 for participants without formal education, 20/21 for those with 1‐6 years of education (primary school), and 24/25 for those with >6 years of education (middle school or higher). The sensitivity and specificity of the cutoff scores of the C‐MMSE were reported to be 85.2% and 92.7% from a study (Zhang et al., 1990). Finally, 269 subjects with cognitive impairment were identified.

### 2.2 Data collection

Data on demographic characteristics and putative risk factors of cognitive impairment were obtained using a standard questionnaire administered by trained staff.

Height and weight were measured with light clothes and without shoes. The height was measured to the nearest 0.5 cm, and the weight to the nearest 0.1 kg. Waist circumference was measured at the level midway between the lower rib margin and the iliac crest. And the measurements of hip circumference were taken around the pelvis at the point of maximal protrusion of the buttocks. Neck circumference was measured with head erect and eyes facing forward, horizontally at the upper margin of the laryngeal prominence (Adam's apple). All the waist, hip, and neck circumferences were measured to the closest 0.1 cm. And each of these measurements was taken by at least two healthcare workers. One took the measurements, and the other recorded the readings. The body mass index (BMI) was calculated as the weight in kilograms divided by the height in meters squared. We evaluated overweight and abdomen obesity according to BMI and waist‐to‐hip ratio (WHR), respectively. Overweight was defined as BMI ≥ 24 (kg/m^2^), and abdomen obesity defined as WHR ≥0.9 for men and WHR ≥0.85 for women. Blood pressure was measured twice after each subject had been seated for 10 min. The average was used for analysis.

Total cholesterol (TC) and triglycerides (TG) were analyzed enzymatically on a Beckman Synchron CX5 Delta Clinical System (Beckman Coulter, Inc, Fullerton, CA, USA) using commercial reagents. Hyperlipemia was defined as TC ≥5.72 mmol/L and/or TG ≥1.69 mmol/L. Fasting plasma glucose (FPG) was examined by a glucose analyzer (Roche, Basel, Switzerland), and hyperglycemia was defined as FPG ≥6.1 mmol/L. Smoking was defined as smoke daily, and alcohol drinking was defined as alcohol consumption at least once per week. Snoring was screened by Berlin questionnaire (Ulasli et al., 2014).

### 2.3 Statistical analysis

Baseline descriptive statistics for the participants were computed for BMI and WHR. Continuous variables were expressed as mean values (*SD*). Categorical variables were expressed as numbers and percentages. BMI and WHR were treated as categorical variables. The participants were also divided into even quartiles according to their NC values, and the quartile NC was also treated as categorical variable. The association between anthropometric measures and cognitive impairment was examined by the logistic regression analysis. Some variables are associated with cognitive impairment and are also associated with NC, BMI, and WHR. These variables (such as age, sex, education level, smoking, alcohol consumption, and snoring) were further adjusted in the multivariate logistic regression models. All statistical analyses were performed using SPSS 20.0 (SPSS for Windows). All *p*‐values were based on two‐sided tests with a significance level of .05.

## 3 RESULTS

In total, 2,500 participants with a mean age of 70.5 years (*SD* = 7.0) and a range from 60 to 94 years were included in this study. There were 1,085 (43.4%) men and 1,415 (56.6%) women, and 269 subjects were identified with cognitive impairment. Among all participants, 483 subjects had WC of 90 cm or greater; 258 subjects had weight of 45 kg or lower. The mean values of NC, BMI, and WHR were 33.01 cm, 24.03 kg/m^2^, and 0.88, respectively. Baseline demographic, anthropometric, and health‐related characteristics, and MMSE scores, groups by NC, BMI, and WHR categories, are shown in Table 1. In this population, for both men and women, the mean age decreased with increasing NC and BMI, while weight, waist, and hip increased with NC, BMI, and WHR. For men, the mean values of SBP and DBP increased with NC, BMI, and WHR. For women, the mean values of SBP increased with NC, BMI, and WHR, and DBP increased with NC, BMI. The percentage of overweight (BMI ≥ 24 kg/m^2^) and abdomen obesity (WHR ≥ 0.9 for men and WHR ≥ 0.85 for women) were both higher in women than in men (*p* = .031 for BMI and *p* < .001 for WHR). The percentage of smokers, alcohol consumers and snorer, and the number of people with at least 1 year of education were all higher in men than in women (all *p*‐values <.001). The mean C‐MMSE score was significantly higher (*p *<* *.001) in men (25.82, *SD*: 3.9) than in women (22.87, *SD*: 4.4). For subjects with the lowest quartile of NC, the mean C‐MMSE score was also the lowest. And it was significantly different (*p* < .05) from the other three quartiles for men, as well as was different (*p* < .05) from the third quartile for women. The C‐MMSE scores were higher in the groups with a BMI ≥ 24 kg/m^2^ for men and a WHR ≤0.85 for women.

**Table 1:** Characteristics of subjects classified by NC, BMI, and WHR

Characteristics	NC (cm)				BMI (kg/m^2^)		WHR	
	Q1 (≤31.0)	Q2 (31.1–33.0)	Q3 (33.1–35.0)	Q4 (≥35.1)	<24	≥24	<0.9	≥0.9
Men								
Number of subjects, *n* (%)	49 (4.5)	223 (20.6)	327 (30.1)	486 (44.8)	580 (53.5)	505 (46.4)	725 (66.8)	360 (33.1)
Age, years (*SD*)	74.3 (6.0)	72.4 (6.9)	70.5 (7.0)	68.9 (5.9)	71.4 (6.8)	69.1 (6.2)	70.5 (6.5)	70.1 (6.8)
Body weight, kg (*SD*)	49.6 (10.1)	52.8 (7.0)	60.2 (6.1)	70.0 (8.5)	55.8 (6.8)	70.5 (8.0)	59.9 (8.9)	68.2 (10.9)
Height, cm (*SD*)	157.9 (5.7)	159.3 (6.0)	161.3 (6.1)	164.1 (5.8)	161.4 (6.3)	162.7 (6.2)	161.6 (6.1)	162.9 (6.5)
Waist, cm (*SD*)	75.1 (10.1)	75.9 (6.8)	81.1 (7.0)	87.3 (7.5)	77.8 (7.1)	88.0 (7.1)	79.2 (7.4)	89.4 (7.1)
Hip, cm (*SD*)	86.6 (8.1)	88.7 (5.8)	92.5 (6.2)	96.8 (6.4)	90.1 (6.2)	97.2 (6.3)	92.5 (7.0)	95.1 (7.1)
Systolic blood pressure, mmHg	125 (19.1)	127 (17.1)	128 (16.4)	131 (16.0)	128 (16.8)	131 (16.3)	128 (16.8)	131 (16.0)
Diastolic blood pressure, mmHg	75 (8.2)	77 (9.4)	78 (9.2)	82 (9.5)	78 (9.3)	81 (9.5)	79 (9.2)	81 (9.9)
Educated for>1 year, *n* (%)	27 (55.1)	131 (58.7)	222 (67.9)	360 (74.1)	206 (35.5)	139 (27.5)	234 (32.3)	111 (30.8)
Snoring, *n* (%)	26 (53.1)	116 (52.0)	180 (30.1)	318 (44.8)	310 (53.4)	330 (65.3)	430 (59.3)	210 (58.3)
Smoking, *n* (%)	23 (46.9)	118 (52.9)	158 (48.3)	225 (46.3)	300 (51.7)	224 (44.4)	362 (49.9)	162 (45.0)
Alcohol consumption, *n* (%)	17 (34.7)	87 (39.0)	129 (39.4)	188 (38.7)	232 (40.0)	189 (37.4)	282 (38.9)	139 (38.6)
C‐MMSE score	23.6 (4.9)	25.0 (4.3)	25.8 (3.5)	26.3 (3.7)	25.3 (4.1)	26.4 (3.6)	25.7 (3.9)	25.9 (3.9)
							<0.85	≥0.85
Women								
Number of subjects	689 (48.7)	442 (31.2)	194 (13.7)	90 (6.4)	695 (49.1)	720 (50.9)	490 (34.6)	925 (65.4)
Age, years, mean (*SD*)	71.7 (7.5)	69.4 (7.0)	69.0 (6.2)	70.5 (6.8)	71.6 (7.4)	69.5 (6.9)	69.7 (6.9)	71.0 (7.4)
Body weight, kg, mean (*SD*)	49.3 (6.7)	57.4 (6.5)	63.5 (6.8)	67.5 (9.2)	48.5 (6.0)	61.1 (7.3)	51.7 (8.0)	56.7 (9.3)
Height, cm, mean (*SD*)	149.4 (6.4)	151.2 (6.0)	151.8 (6.0)	153.2 (6.7)	150.6 (6.5)	150.5 (6.2)	151.2 (6.2)	150.2 (6.4)
Waist, cm, mean (*SD*)	75.9 (7.6)	82.4 (7.2)	87.7 (7.5)	90.9 (7.5)	75.0 (7.1)	85.9 (7.3)	73.3 (6.6)	84.4 (7.7)
Hip, cm, mean (*SD*)	88.8 (6.6)	94.1 (6.2)	98.3 (6.6)	100.0 (8.5)	88.2 (6.3)	96.6 (6.6)	90.4 (6.8)	93.6 (7.9)
Systolic blood pressure, mmHg	132 (18.2)	133 (16.8)	136 (17.8)	137 (19.5)	132 (18.0)	134 (17.7)	130 (8.7)	135 (17.3)
Diastolic blood pressure, mmHg	78 (9.5)	80 (9.5)	81 (8.1)	81 (9.1)	78 (8.7)	80.6 (9.8)	78 (9.1)	80 (9.4)
Educated for >1 year, *n* (%)	164 (23.8)	115 (26.0)	56 (28.9)	23 (25.6)	160 (23.0)	198 (27.5)	142 (29.0)	216 (23.4)
Snoring, *n* (%)	316 (45.9)	214 (48.4)	118 (60.8)	49 (54.4)	329 (47.3)	368 (51.1)	236 (48.2)	461 (49.8)
Smoking, *n* (%)	1 (0.1)	4 (0.9)	4 (2.1)	4 (4.4)	7 (1.0)	6 (0.8)	3 (0.6)	10 (1.1)
Alcohol consumption, *n* (%)	1 (0.1)	1 (0.2)	2 (1.0)	5 (5.6)	5 (.07)	4 (0.6)	2 (0.4)	7 (0.8)
C‐MMSE score	22.5 (4.6)	23.0 (4.1)	23.5 (4.2)	22.8 (3.8)	22.6 (4.5)	23.0 (4.2)	23.3 (4.2)	22.6 (4.4)

Q1 = the lowest quartile of NC, Q2 = the second quartile of NC, Q3 = the third quartile of NC, and Q4 = the highest quartile of NC. NC, neck circumference; BMI, body mass index; WHR, waist‐to‐height ratio.

Table 2 presents the unadjusted association of cognitive impairment with age, gender, cigarette smoking, alcohol drinking, TC ≥ 5.72 mmol/L, TG ≥1.69 mmol/L, FPG ≥6.1 mmol/L, high blood pressure, BMI, WHR, and NC. Higher age, women, higher NC (both males and females), higher WHR, TC ≥5.72 mmol/L, and snoring were associated with the increased risk of cognitive impairment. However, significant associations were not observed for TG ≥1.69 mmol/L, FPG ≥6.1 mmol/L, cigarette smoking, alcohol drinking, without formal education, or high blood pressure with cognitive impairment. These associations were further evaluated by multivariate logistic regression analysis.

**Table 2:** Crude association of cognitive impairment with relevant variables and NC, BMI, and WHR

Variable	No	Odds ratio (95% confidence interval)	*p*‐Value
Age group, years			
60–69	1,266	1.0	
70–79	908	4.55 (3.21–6.44)	**<.001**
80 and above	326	10.11 (6.90–14.81)	**<.001**
Gender			
Men	1,085	1.0	
Women	1,415	2.05 (1.55–2.71)	**.037**
NC (both males and females)			
Q1	738	1.0	
Q2	665	1.64 (1.10–2.44)	**.015**
Q3	521	2.00 (1.34–3.00)	**.001**
Q4	576	3.24 (2.23–4.69)	**<.001**
BMI (kg/m^2^)			
<24	1,275	1.0	
≥24	1,225	1.54 (1.11–2.15)	**.010**
WHR			
<0.85/0.90	1,215	1.0	
≥0.85/0.90	1,285	1.44 (1.11–1.86)	**.017**
TC ≥ 5.72 mmol/L			
No	2,084	1.0	
Yes	416	1.51 (1.08–2.12)	**.016**
TG ≥ 1.69 mmol/L			
No	2,003	1.0	
Yes	497	0.93 (0.67–1.29)	.642
Blood pressure			
<140/90 mmHg	1,467	1.0	
≥140/90 mmHg	1,033	1.015 (0.78–1.31)	.911
FPG ≥ 6.1 mmol/L			
No	2,050	1.0	
Yes	450	0.98 (0.71–1.37)	.944
Educational level			
Formal education	1,098	1.0	
Without formal education	1,402	0.75 (0.55–1.02)	.069
Smoking			
No	1,963	1.0	
Yes	537	1.10 (0.74–1.61)	.629
Alcohol consumption			
No	2,070	1.0	
Yes	430	1.01 (0.67–1.28)	.970
Snoring			
No	1,163	1.0	
Yes	1,337	2.08 (1.56–2.75)	**<.001**

Q1 = the lowest quartile of NC, Q2 = the second quartile of NC, Q3 = the third quartile of NC, and Q4 = the highest quartile of NC. NC, neck circumference; BMI, body mass index; WHR, waist‐to‐height ratio; TC, total cholesterol; TG, triglycerides; FPG, fasting plasma glucose.

The multivariable‐adjusted associations between cognitive impairment and relevant variables are presented in Table 3. The association between higher BMI, WHR groups, and cognitive impairment still differed remarkably with adjustment for age group, gender, education level, smoking, alcohol consumption, and snoring. And higher TC was significantly (*p *=* *.015) associated with cognitive impairment. Compared to the lowest quartile of NC, the second quartile, the third quartile, and the highest quartile were significantly associated with cognitive impairment (for males and females, respectively) after being adjusted for age group, education level, smoking, alcohol consumption, and snoring. However, no significant associations were observed for higher blood pressure, FPG, and TG.

**Table 3:** Results of multivariate logistic regression analysis for variables associated with cognitive impairment

Variable	No	Odds ratio (95% confidence interval)	*p*‐Value
NC (males)			
Q1	272	1.0	
Q2	327	4.73 (2.18–10.28)	**<.001**
Q3	236	4.09 (1.82–9.21)	**.001**
Q4	250	7.84 (3.63–16.95)	**<.001**
NC (females)			
Q1	408	1.0	
Q2	306	0.86 (0.46–1.62)	.650
Q3	417	2.37 (1.46–3.85)	**<.001**
Q4	284	2.77 (1.66–4.60)	**<.001**
BMI (kg/m^2^)			
<24	1,275	1.0	
≥24	1,225	1.55 (1.11–2.16)	**.010**
WHR			
<0.85/0.90	1,215	1.0	
≥0.85/0.90	1,285	1.44 (1.07–1.95)	**.017**
Blood pressure			
<140/90 mmHg	1,467	1.0	
≥140/90 mmHg	1,033	0.94 (0.72–1.22)	.630
FPG ≥ 6.1 mmol/L			
No	2,050	1.0	
Yes	450	1.00 (0.72–1.39)	.994
TC ≥ 5.72 mmol/L			
No	2,084	1.0	
Yes	416	1.52 (1.09–2.13)	**.015**
TG ≥ 1.69 mmol/L			
No	2,003	1.0	
Yes	497	0.92 (0.66–1.27)	.610

Q1 = the lowest quartile of NC, Q2 = the second quartile of NC, Q3 = the third quartile of NC, and Q4 = the highest quartile of NC. NC, neck circumference; BMI, body mass index; WHR, waist‐to‐height ratio; TC, total cholesterol; TG, triglycerides; FPG, fasting plasma glucose.

Multivariate logistic regression models with adjustment for age group (60–69, 70–79, and ≥80 years), gender, education level (formal education, without formal education), smoking (no, yes), alcohol consumption (no, yes), and snoring (no, yes).

Results from the interaction analysis between TC and a few other variables (NC, BMI, and WHR) to the risk of cognitive impairment are presented in Table 4. High TC was significantly associated with increased prevalence of cognitive impairment in higher BMI and WHR groups after controlling for confounders. In quartile of both males’ and females’ NC groups, high TC was significantly interacted to cognitive impairment, excepting the first quartile of males’ NC. A test of interaction between high TC and the quartiles of NC groups for both males and females (except for the first quartile of males’ NC group, all *p* < .05), BMI (*p* < .001), or WHR (*p* = .007) was statistically significant from the log likelihood ratio tests.

**Table 4:** Interactions between TC and other variables (NC, BMI, and WHR) for the risk of cognitive impairment

Variable	TC ≥ 5.72 mmol/L	Subjects (number)	Cognitive impairment		
			OR	95% confidence interval	*p*‐Value
NC (males)					
Q1	No	245	1.0		
	Yes	27	0.78	0.08–7.13	.826
Q2	No	297	1.0		
	Yes	31	3.34	1.43–7.98	**.005**
Q3	No	214	1.0		
	Yes	21	3.46	1.13–10.61	**.029**
Q4	No	226	1.0		
	Yes	24	2.62	1.02–6.70	**.044**
NC (females)					
Q1	No	314	1.0		
	Yes	94	2.52	1.08–5.89	**.033**
Q2	No	229	1.0		
	Yes	77	3.74	1.36–10.28	**.011**
Q3	No	335	1.0		
	Yes	82	9.13	4.69–17.74	**<.001**
Q4	No	221	1.0		
	Yes	63	2.34	1.14–4.79	**.020**
BMI (kg/m^2^)					
<24	No	1,079	1		
	Yes	196	0.504	0.226–1.126	.095
≥24	No	1,005	1		
	Yes	220	2.009	1.372–2.942	**<.001**
WHR					
<0.85/0.90	No	1,052	1		
	Yes	163	0.881	0.441–1.761	.720
≥0.85/0.90	No	1,032	1		
	Yes	253	1.699	1.155–2.498	**.007**

Multivariate logistic regression models with adjustment for age group (60–69, 70–79, and ≥80 years), education level (formal education, without formal education), smoking (no, yes), alcohol consumption (no, yes), and snoring (no, yes). NC, neck circumference; BMI, body mass index; WHR, waist‐to‐height ratio; TC, total cholesterol.

## 4 DISCUSSION

NC is a simple and time‐saving screening measure, which can be used as an index of upper body fat distribution to identify obesity and to predict obesity‐related metabolic abnormality (Preis et al., 2010; Stabe et al., 2013; Yan et al., 2014). In this cross‐sectional study of an older population from Shanghai suburb, higher NC and an upper body fat depot, as well as higher BMI and higher WHR, were positively associated with an increased prevalence of cognitive impairment. These results suggest that the effects of upper body fat, just as central obesity, may be a risk factor for cognitive impairment.

Obesity is an epidemic that associated with adverse health outcomes, such as hypertension, diabetes, stroke, and sleep apnea (Mellendijk, Wiesmann, & Kiliaan, 2015; Shin et al., 2014). Obesity and Mets are associated with vascular risk factors, which in turn may increase dementia risk. Kharabian et al. (2016) assessed the effects of BMI on gray matter volume and cognition in community‐dwelled older adults and found that a higher BMI in older adults was associated with widespread gray matter alterations. Pasha et al. (2017) used magnetic resonance imaging (MRI) scan to quantify white matter disease and cognitive test battery to evaluate the cognitive function in 126 adults aged 40–62 years and concluded obesity at midlife affected cerebral white matter prior to detectable cognitive changes. In animal studies, high‐fat diet had been shown to impair hippocampal neurogenesis and influence the learning performance (Klein et al., 2016), which is in accordance with the findings by Pasha (Pasha et al., 2017) and Kharabian (Kharabian et al., 2016). Our results, which higher NC, higher BMI, and WHR were associated with the increased prevalence of cognitive impairment, were consistent with the results from aforementioned studies (Kharabian et al., 2016; Liu et al., 2015). Higher NC among elderly is a risk factor for cognitive impairment.

Our results that weight, waist, and hip increased with NC, BMI, and WHR among both men and women (data shown in Table 1) suggested that high NC, high BMI, and WHR were associated with overweight or obesity. Li et al. (2014) investigated 177 cases of Chinese patients aged 35–75 years and found that NC correlated significantly with abdominal visceral adipose tissue, especially in males. It may account for the fact that neck fat area is associated with abdominal visceral fat. Another cross‐sectional survey of population‐based data among 2,092 cases of elderly Chinese (Yan et al., 2014) showed that NC was strongly associated with BMI and WC, and NC was significantly independent indicators for obesity. Joshipura, Munoz‐Torres, Vergara, Palacios, & Perez (2016) investigated 1,206 participants of overweight or obesity aged from 40 to 65 years and found the highest (compared to the lowest) tertile had a higher association with Mets. Although these studies carried in different populations (including young adults, elderly people, and population of overweight or obesity), the results were coincident. Our findings were in agreement with these studies (Joshipura et al., 2016; Li et al., 2014; Yan et al., 2014), in which showed NC was associated with overweight or obesity. It was conferred that NC might be a useful tool for identifying Mets and obesity. However, there were incompatible results. Pedditizi, Peters, & Beckett (2016) performed a systematic review and meta‐analysis regarding possible association of overweight or obesity and the subsequent development of dementia. For all the studies, the age over 65 years was defined as late life, while below 65 as midlife. Being obese in midlife had a positive association with the incidence of dementia, but the opposite was seen in the late life. These results were heterogeneous and the explanation for that might be due to differences in methodology, for example, differences in study designs, participants, and measurements.

Our results indicated that high TC was associated with a high risk of cognitive impairment. Studies have shown that elevated plasma cholesterol levels serve as a risk factor for cognitive decline (Hohsfield, Daschil, Oradd, Stromberg, & Humpel, 2014; Park et al., 2013; Sharma & Taliyan, 2014). Hohsfield et al. (2014) investigated the effects of high‐cholesterol diet on AD‐related neuropathology, cognitive function, neuroinflammation, and cerebrovascular changes in wild‐type and transgenic AD mice at various ages and stages of disease. They found that hypercholesterolemia could alter cognitive function, AD‐related pathology in WT, and triple‐transgenic AD mice depending on age and stage of disease. Park et al. (2013) subjected the mice to a water maze to verify whether hypercholesterolemia had effects on cognitive impairment. It was observed that hypercholesterolemia significantly exacerbated the cognitive decline when compared to the Aβ_25–35_‐injected group of normal mice at 4 weeks after Aβ injection. It seems that high TC could promote increased cerebrovascular and blood–brain barrier damage, ultimately leading to the influx of neurotoxic molecules and enhanced inflammatory processes, followed by alterations in AD pathology and the initiation of neurodegeneration and cognitive deficits. Our data were compatible with the aforementioned studies (Hohsfield et al., 2014; Park et al., 2013; Sharma & Taliyan, 2014). However, Benito‐Leon, Vega‐Quiroga, Villarejo‐Galende, & Bermejo‐Pareja (2015) reported discrepant outcomes. They investigated a prospective population‐based cohort in 2,015 cases of elders and found cognitive test scores among elders with hypercholesterolemic declined more slowly than observed in their nonhypercholesterolemic counterparts. The explanation for the inconsistent results might be due to different study designs. Further studies are needed in this regard.

In our study, high TC was also significantly associated with the increased prevalence of cognitive impairment in higher BMI and WHR groups. It is well known that cholesterol is an essential substance for maintaining normal structure and function of the brain. But unfortunately, a long‐term high‐cholesterol diet can lead to a variety of pathological changes of the brain such as Aβ accumulation, reactive gliosis, neuroinflammation, neuronal death, and synaptic degeneration (Asadbegi, Yaghmaei, Salehi, Ebrahim‐Habibi, & Komaki, 2016; Klein et al., 2016). BMI and WHR have been proposed widely as frequently used methods of the indices of obesity (Pedditizi et al., 2016). NC has not been given as much consideration as BMI and WHR, which was initially found to be correlated with obstructive sleep syndrome (Davies & Stradling, 1990) and recently was recognized as a screening measure for identifying overweight, obesity, and Mets (Preis et al., 2010; Stabe et al., 2013; Yan et al., 2014). In our study, when NC or BMI or WHR increased with high TC, the ORs for cognitive impairment increased, which suggested that the high TC could play a synergistic interaction with NC or BMI or WHR in the development and presence of cognitive impairment. A higher NC may result from the upper fat depot and may confer an increased risk of cognitive impairment in the elderly population.

Many studies have shown a positive correlation between high blood pressure and cognitive function (Perrotta, Lembo, & Carnevale, 2016), and hypertension could generate small vessel disease, which was a major cause of vascular cognitive impairment. High TG was reported to be associated with cognitive impairment and diabetes, associated with microvascular complications, was also closely related to cognitive dysfunction (Umegaki et al., 2012). Umegaki et al. (Umegaki et al., 2012) carried an observational study and found hypertension and hypertriglyceridemia at baseline were associated with more than 5‐point declines in MMSE. Although high blood pressure, high TG, and high FPG are key components of metabolic syndrome, no significant associations were observed with increased cognitive impairment from our study. The inconsistency may be, in part, due to certain reasons. In this study, the number of subjects with a history of hypertension, hypertriglyceridemia, and diabetes was small. This might be due to that our participants were mostly farmers and with low education level. There is a certain difference on diet habit of these individuals compared to those living in urban areas (Cui et al., 2013). Moreover, because adherence to obesity is a risk of cognitive impairment, the true association between obesity and cognitive decline may have been obscured by residual confounding.

There are some limitations. For example, a cross‐sectional survey study could not finally make a conclusion regarding the causal relationship between NC and cognitive impairment. NC is a screening measure for identifying overweight and obesity. Although our epidemiological study found NC was associated with cognitive impairment, our findings were yet to be verified in other communities or other larger populations. Therefore, the generalization of our finding should be cautious. Unfortunately, in this study, we did not make further investigations for the diagnosis of dementia as well as its subtypes such as Alzheimer's disease or vascular dementia due to the feasibility and other conditions, so that we are not able to provide more information regarding those aspects.

In summary, our results indicated that increased NC, BMI, and WHR were significantly associated with the increased risk of cognitive impairment and there were positive interactions between TC and NC, between TC and BMI, or between TC and WHR on cognitive impairment among elderly.

## CONFLICT OF INTERESTS

The authors declare that they have no conflict of interests.
